# Ethyl (*E*)-3-(6-methyl-4-oxo-4*H*-chromen-3-yl)prop-2-enoate

**DOI:** 10.1107/S1600536812038585

**Published:** 2012-09-19

**Authors:** Sammer Yousuf, Asma Mukhtar, Nida Ambreen, Syed Muhammad Saad, Khalid M. Khan

**Affiliations:** a H.E.J. Research Institute of Chemistry, International Center for Chemical and Biological Sciences, University of Karachi, Karachi 75270, Pakistan

## Abstract

In the title compound, C_15_H_14_O_4_, the chromone ring system is close to being planar [maximum deviation = 0.015 (2) Å]. The double bond of the ethyl prop-2-enoate chain adopts an *E* conformation and an intra­molecular C—H⋯O hydrogen bond generates an *S*6 ring. In the crystal, inversion dimers linked by pairs of C—H⋯O hydrogen bonds generate *R*_2_^2^(14) loops. Weak π–π inter­actions [centroid–centroid distance = 3.8493 (12) Å] also occur.

## Related literature

For the biological activity of chromones, see: Patel *et al.* (2011 ▶); Khan *et al.* (2010 ▶); Gautam *et al.* (2010 ▶). For a related structure, see: Wang & Kong (2007 ▶).
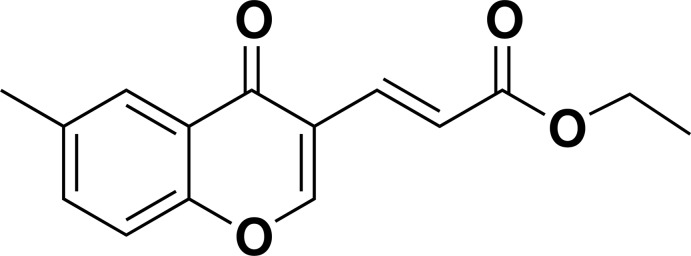


## Experimental

### 

#### Crystal data

C_15_H_14_O_4_
*M* *_r_* = 258.26
Monoclinic, 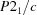

*a* = 13.8663 (12) Å
*b* = 12.3512 (10) Å
*c* = 7.6947 (6) Å
β = 96.390 (2)°
*V* = 1309.65 (19) Å^3^
*Z* = 4
Mo *K*α radiation
μ = 0.10 mm^−1^
*T* = 298 K
0.34 × 0.25 × 0.16 mm

#### Data collection

Bruker SMART APEX CCD diffractometer
Absorption correction: multi-scan (*SADABS*; Bruker, 2000 ▶) *T*_min_ = 0.968, *T*_max_ = 0.985
7621 measured reflections
2431 independent reflections
1650 reflections with *I* > 2σ(*I*)
*R*_int_ = 0.027

#### Refinement

*R*[*F* ^2^ > 2σ(*F* ^2^)] = 0.048
*wR*(*F* ^2^) = 0.142
*S* = 1.04
2431 reflections
175 parameters
H-atom parameters constrained
Δρ_max_ = 0.17 e Å^−3^
Δρ_min_ = −0.15 e Å^−3^

### 

Data collection: *SMART* (Bruker, 2000 ▶); cell refinement: *SAINT* (Bruker, 2000 ▶); data reduction: *SAINT*; program(s) used to solve structure: *SHELXS97* (Sheldrick, 2008 ▶); program(s) used to refine structure: *SHELXL97* (Sheldrick, 2008 ▶); molecular graphics: *SHELXTL* (Sheldrick, 2008 ▶); software used to prepare material for publication: *SHELXTL* and *PARST* (Nardelli, 1995 ▶) and *PLATON* (Spek, 2009 ▶).

## Supplementary Material

Crystal structure: contains datablock(s) global, I. DOI: 10.1107/S1600536812038585/hb6950sup1.cif

Structure factors: contains datablock(s) I. DOI: 10.1107/S1600536812038585/hb6950Isup2.hkl

Supplementary material file. DOI: 10.1107/S1600536812038585/hb6950Isup3.cml

Additional supplementary materials:  crystallographic information; 3D view; checkCIF report

## Figures and Tables

**Table 1:** Hydrogen-bond geometry (Å, °)

*D*—H⋯*A*	*D*—H	H⋯*A*	*D*⋯*A*	*D*—H⋯*A*
C11—H11*A*⋯O2	0.93	2.28	2.908 (2)	124
C9—H9*A*⋯O3^i^	0.93	2.37	3.276 (3)	164

Symmetry code: (i) 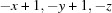.

**Fig. 1.**
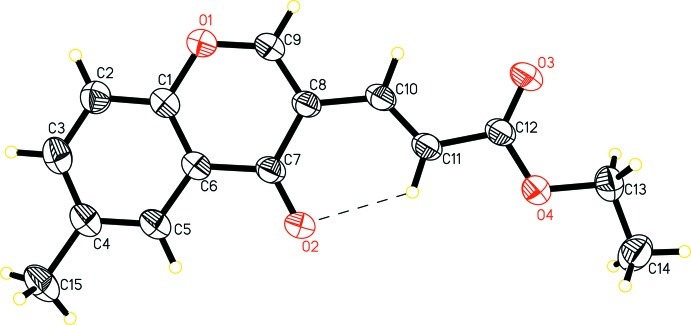
The molecular structure of (I) with displacement ellipsoids drawn at 30% probability level.

**Fig. 2.**
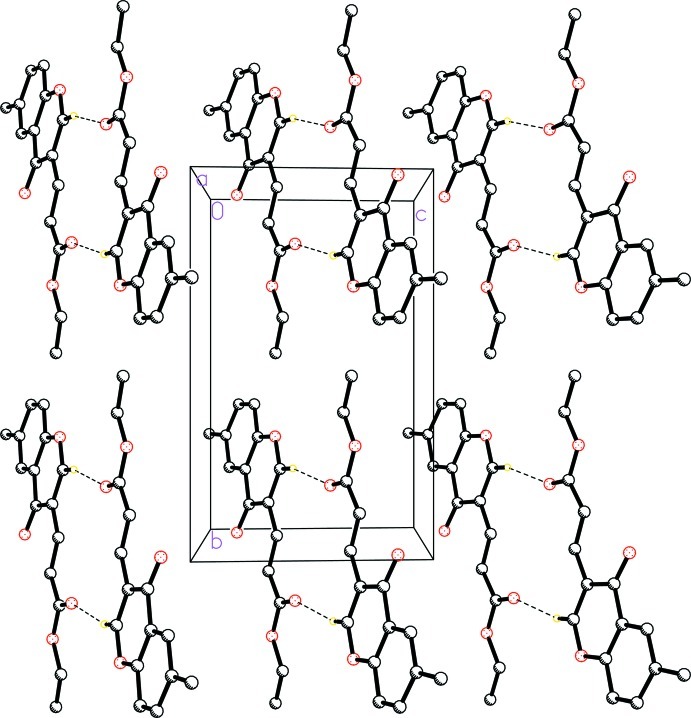
The crystal packing of the title compound I.

## supplementary crystallographic information

### Comment

Chromone is a group of naturally occurring oxygen containing heterocyclic compounds having a benzene ring fused with pyran ring. They are widely distributed in plant kingdom and form the basic nucleus of important compounds such as anthocyanin and flavonoids. The chromone moiety forms an important component of pharmacophores of a number of biologically active molecules of synthetic as well as natural origin and therefore responsible for various biological activities (e.g. Patel *et al.* (2011); Gautam *et al.* (2010); Khan *et al.* (2010). The title compound is a chromone derivative obtained as a part of our ongoing project to synthesize libraries of chromone derivatives in order to study their different biological activities. The structure of title compound (Fig. 1) is composed of almost planner chromone moiety (O1/C1–C9) with maximum deviation of 0.015 (2) Å for C7 atom from the root mean square plane. The C11–C12 (1.462 (3) Å) olefinic bond of ethyl prop-2-enoate chain (O3–O4/C10–C14) attached to chromone moiety adopt an *E* configuartion. The shorter bond lengths of C11–C12 = 1.462 (3) Å than the expected C–C single bond length is due to the conjugation effects of the olefinc bond (C11–C12, 1.462 (3) Å) with carbonyl carbon (O3/C12) of ethyl prop-2-enoate chain (O3–O4/C10–C14). The bond lengths and angle were found to be similar as in structurally realted compound (Wang & Kong, 2007). The *E* conformation of olefinic bound further stabilized by an intramolecular C11–C11A···O2 intramolecular hydrogen bond. In the crystal inversion-related molecules are consolidated by C9–C9A···O3 hydrogen bond and found stacked along the *a*-axis. The crystal structure also features weak π–π interaction between pyrane (*Cg*(1)= O1/C6–C9) and benzene (*Cg*(2)= C1–C6) of chromone moeity ((*Cg*(1)to *Cg*(2) distance = 3.8493 (12) Å;*X*,1/2-Y,-1/2+*Z*)

### Experimental

A mixture of 3-formyl chromone (10 mmol) and malonic acid (20 mmol), using pyridine (15 ml) as solvent was refluxed in 500 mL round-bottomed flask for 30–45 minutes with vigorous stirring. After completion of reaction (monitored by TLC), the reaction mixture was cooled to room temperature, acidified by concntrated hydrochloric acid (pH 1.0) and stirred again for 30 minutes at room temprature. The yellow colored solid (1.02 g) obtained was filtered and washed with water. The crude residue was dried, dissolved in ethanol (50 ml) along with few drops of H_2_SO_4_ and refluxed for 24 h (progress of the reaction was monitored by TLC). After completion of reaction the solvent was evaporated under vacuum followed by addition of saturated solution of NaHCO_3_ and extracted with ethyl acetate, washed with water. The organic phase was dried over Na_2_SO_4_. The solvent was evaporated under reduced pressure to obtain crude product which was further recrystallized in ethanol to obtain yellow blocks in 82% yield (0.94 g).

### Refinement

H atoms on Methyl, methylene and methine were positioned geometrically with C—H = 0.96 Å (CH_3_), 0.97 Å (CH_3_) and 0.93 Å (CH) and constrained to ride on their parent atoms with *U*_iso_(H)= 1.5*U*_eq_(CH_3_) and 1.2*U*_eq_(CH_2_, CH).

### Crystal data

**Table:**

C_15_H_14_O_4_	*F*(000) = 544
*M**_r_* = 258.26	*D*_x_ = 1.310 Mg m^−^^3^
Monoclinic, *P*2_1_/*c*	Mo *K*α radiation, λ = 0.71073 Å
*a* = 13.8663 (12) Å	Cell parameters from 1626 reflections
*b* = 12.3512 (10) Å	θ = 3.0–23.3°
*c* = 7.6947 (6) Å	µ = 0.10 mm^−^^1^
β = 96.390 (2)°	*T* = 298 K
*V* = 1309.65 (19) Å^3^	Block, colourless
*Z* = 4	0.34 × 0.25 × 0.16 mm

### Data collection

**Table:**

Bruker SMART APEX CCD diffractometer	2431 independent reflections
Radiation source: fine-focus sealed tube	1650 reflections with *I* > 2σ(*I*)
Graphite monochromator	*R*_int_ = 0.027
ω scan	θ_max_ = 25.5°, θ_min_ = 2.2°
Absorption correction: multi-scan (*SADABS*; Bruker, 2000)	*h* = −16→16
*T*_min_ = 0.968, *T*_max_ = 0.985	*k* = −14→14
7621 measured reflections	*l* = −9→9

### Refinement

**Table:**

Refinement on *F*^2^	Secondary atom site location: difference Fourier map
Least-squares matrix: full	Hydrogen site location: inferred from neighbouring sites
*R*[*F*^2^ > 2σ(*F*^2^)] = 0.048	H-atom parameters constrained
*wR*(*F*^2^) = 0.142	*w* = 1/[σ^2^(*F*_o_^2^) + (0.0754*P*)^2^ + 0.0649*P*] where *P* = (*F*_o_^2^ + 2*F*_c_^2^)/3
*S* = 1.04	(Δ/σ)_max_ < 0.001
2431 reflections	Δρ_max_ = 0.17 e Å^−^^3^
175 parameters	Δρ_min_ = −0.15 e Å^−^^3^
0 restraints	Extinction correction: *SHELXTL* (Sheldrick, 2008), Fc^*^=kFc[1+0.001xFc^2^λ^3^/sin(2θ)]^-1/4^
Primary atom site location: structure-invariant direct methods	Extinction coefficient: 0.0028 (16)

### Special details

**Table:**

Geometry. All e.s.d.'s (except the e.s.d. in the dihedral angle between two l.s. planes) are estimated using the full covariance matrix. The cell e.s.d.'s are taken into account individually in the estimation of e.s.d.'s in distances, angles and torsion angles; correlations between e.s.d.'s in cell parameters are only used when they are defined by crystal symmetry. An approximate (isotropic) treatment of cell e.s.d.'s is used for estimating e.s.d.'s involving l.s. planes.
Refinement. Refinement of *F*^2^ against ALL reflections. The weighted *R*-factor *wR* and goodness of fit *S* are based on *F*^2^, conventional *R*-factors *R* are based on *F*, with *F* set to zero for negative *F*^2^. The threshold expression of *F*^2^ > σ(*F*^2^) is used only for calculating *R*-factors(gt) *etc*. and is not relevant to the choice of reflections for refinement. *R*-factors based on *F*^2^ are statistically about twice as large as those based on *F*, and *R*- factors based on ALL data will be even larger.

### Fractional atomic coordinates and isotropic or equivalent isotropic displacement parameters (Å^2^)

**Table:**

	*x*	*y*	*z*	*U*_iso_*/*U*_eq_	
O1	0.74141 (9)	0.20992 (10)	0.17694 (19)	0.0713 (4)	
O2	0.85161 (10)	0.49847 (11)	0.3624 (2)	0.0786 (5)	
O3	0.51893 (10)	0.66244 (12)	0.0798 (2)	0.0880 (5)	
O4	0.63447 (9)	0.77677 (10)	0.18750 (18)	0.0677 (4)	
C1	0.83451 (14)	0.21480 (15)	0.2624 (3)	0.0597 (5)	
C2	0.88511 (15)	0.11889 (17)	0.2813 (3)	0.0715 (6)	
H2A	0.8566	0.0542	0.2405	0.086*	
C3	0.97837 (16)	0.12068 (19)	0.3615 (3)	0.0731 (6)	
H3A	1.0133	0.0563	0.3724	0.088*	
C4	1.02265 (14)	0.21586 (18)	0.4273 (3)	0.0653 (6)	
C5	0.97015 (13)	0.31040 (17)	0.4062 (2)	0.0614 (5)	
H5A	0.9987	0.3749	0.4476	0.074*	
C6	0.87513 (12)	0.31198 (15)	0.3241 (2)	0.0540 (5)	
C7	0.81876 (12)	0.41241 (16)	0.3029 (2)	0.0570 (5)	
C8	0.72174 (12)	0.40105 (15)	0.2076 (2)	0.0537 (5)	
C9	0.69141 (14)	0.30212 (16)	0.1524 (3)	0.0637 (5)	
H9A	0.6298	0.2975	0.0913	0.076*	
C10	0.65611 (13)	0.49171 (15)	0.1662 (2)	0.0571 (5)	
H10A	0.5964	0.4750	0.1047	0.069*	
C11	0.67129 (13)	0.59483 (16)	0.2054 (3)	0.0594 (5)	
H11A	0.7292	0.6151	0.2697	0.071*	
C12	0.59924 (13)	0.67789 (15)	0.1500 (3)	0.0595 (5)	
C13	0.57176 (15)	0.86723 (17)	0.1372 (3)	0.0764 (6)	
H13A	0.5168	0.8677	0.2049	0.092*	
H13B	0.5476	0.8624	0.0142	0.092*	
C14	0.63063 (19)	0.96782 (18)	0.1716 (3)	0.0915 (8)	
H14A	0.5895	1.0300	0.1511	0.137*	
H14B	0.6807	0.9700	0.0950	0.137*	
H14C	0.6596	0.9680	0.2909	0.137*	
C15	1.12484 (15)	0.2147 (2)	0.5173 (3)	0.0877 (7)	
H15A	1.1556	0.2827	0.4985	0.132*	
H15B	1.1606	0.1572	0.4703	0.132*	
H15C	1.1234	0.2035	0.6404	0.132*	

### Atomic displacement parameters (Å^2^)

**Table:**

	*U*^11^	*U*^22^	*U*^33^	*U*^12^	*U*^13^	*U*^23^
O1	0.0564 (9)	0.0616 (9)	0.0918 (11)	−0.0011 (6)	−0.0101 (7)	−0.0061 (7)
O2	0.0551 (8)	0.0610 (9)	0.1124 (12)	−0.0051 (6)	−0.0225 (8)	−0.0025 (8)
O3	0.0525 (9)	0.0756 (10)	0.1274 (14)	−0.0032 (7)	−0.0270 (9)	0.0082 (9)
O4	0.0541 (8)	0.0600 (9)	0.0857 (10)	0.0022 (6)	−0.0068 (7)	0.0026 (7)
C1	0.0510 (11)	0.0680 (13)	0.0589 (12)	0.0022 (9)	0.0009 (9)	0.0014 (10)
C2	0.0715 (14)	0.0655 (13)	0.0760 (14)	0.0070 (10)	0.0019 (11)	−0.0045 (11)
C3	0.0705 (14)	0.0801 (15)	0.0680 (14)	0.0231 (11)	0.0049 (11)	0.0014 (11)
C4	0.0547 (12)	0.0860 (15)	0.0544 (12)	0.0135 (10)	0.0027 (9)	−0.0025 (10)
C5	0.0481 (11)	0.0766 (14)	0.0585 (12)	0.0038 (9)	0.0016 (9)	−0.0031 (10)
C6	0.0450 (10)	0.0643 (12)	0.0521 (11)	0.0008 (8)	0.0023 (8)	0.0018 (9)
C7	0.0440 (10)	0.0626 (12)	0.0628 (12)	−0.0046 (9)	−0.0012 (9)	0.0042 (10)
C8	0.0434 (10)	0.0589 (11)	0.0573 (11)	−0.0043 (8)	−0.0004 (8)	0.0041 (9)
C9	0.0471 (11)	0.0678 (13)	0.0732 (14)	−0.0010 (9)	−0.0067 (10)	0.0017 (10)
C10	0.0416 (10)	0.0668 (13)	0.0611 (12)	−0.0055 (8)	−0.0028 (8)	0.0088 (9)
C11	0.0452 (10)	0.0651 (13)	0.0651 (12)	−0.0041 (9)	−0.0057 (9)	0.0069 (10)
C12	0.0444 (11)	0.0650 (13)	0.0669 (13)	−0.0038 (9)	−0.0036 (9)	0.0065 (10)
C13	0.0689 (14)	0.0685 (14)	0.0901 (16)	0.0141 (10)	0.0014 (12)	0.0090 (11)
C14	0.117 (2)	0.0648 (14)	0.0893 (17)	0.0040 (13)	−0.0039 (15)	−0.0030 (12)
C15	0.0587 (13)	0.118 (2)	0.0827 (16)	0.0251 (13)	−0.0066 (11)	−0.0078 (14)

### Geometric parameters (Å, º)

**Table:**

O1—C9	1.336 (2)	C7—C8	1.465 (2)
O1—C1	1.383 (2)	C8—C9	1.346 (3)
O2—C7	1.225 (2)	C8—C10	1.456 (2)
O3—C12	1.198 (2)	C9—H9A	0.9300
O4—C12	1.335 (2)	C10—C11	1.320 (3)
O4—C13	1.442 (2)	C10—H10A	0.9300
C1—C2	1.376 (3)	C11—C12	1.462 (3)
C1—C6	1.387 (3)	C11—H11A	0.9300
C2—C3	1.370 (3)	C13—C14	1.494 (3)
C2—H2A	0.9300	C13—H13A	0.9700
C3—C4	1.395 (3)	C13—H13B	0.9700
C3—H3A	0.9300	C14—H14A	0.9600
C4—C5	1.376 (3)	C14—H14B	0.9600
C4—C15	1.506 (3)	C14—H14C	0.9600
C5—C6	1.396 (3)	C15—H15A	0.9600
C5—H5A	0.9300	C15—H15B	0.9600
C6—C7	1.466 (3)	C15—H15C	0.9600
			
C9—O1—C1	118.19 (15)	C8—C9—H9A	116.9
C12—O4—C13	117.10 (15)	C11—C10—C8	127.74 (18)
C2—C1—O1	116.79 (18)	C11—C10—H10A	116.1
C2—C1—C6	121.72 (19)	C8—C10—H10A	116.1
O1—C1—C6	121.49 (16)	C10—C11—C12	121.59 (18)
C3—C2—C1	118.6 (2)	C10—C11—H11A	119.2
C3—C2—H2A	120.7	C12—C11—H11A	119.2
C1—C2—H2A	120.7	O3—C12—O4	122.87 (18)
C2—C3—C4	122.18 (19)	O3—C12—C11	126.20 (18)
C2—C3—H3A	118.9	O4—C12—C11	110.92 (16)
C4—C3—H3A	118.9	O4—C13—C14	107.19 (18)
C5—C4—C3	117.80 (19)	O4—C13—H13A	110.3
C5—C4—C15	121.4 (2)	C14—C13—H13A	110.3
C3—C4—C15	120.81 (19)	O4—C13—H13B	110.3
C4—C5—C6	121.70 (19)	C14—C13—H13B	110.3
C4—C5—H5A	119.1	H13A—C13—H13B	108.5
C6—C5—H5A	119.1	C13—C14—H14A	109.5
C1—C6—C5	118.00 (17)	C13—C14—H14B	109.5
C1—C6—C7	120.19 (17)	H14A—C14—H14B	109.5
C5—C6—C7	121.81 (17)	C13—C14—H14C	109.5
O2—C7—C8	123.54 (17)	H14A—C14—H14C	109.5
O2—C7—C6	121.42 (16)	H14B—C14—H14C	109.5
C8—C7—C6	115.04 (17)	C4—C15—H15A	109.5
C9—C8—C10	117.61 (16)	C4—C15—H15B	109.5
C9—C8—C7	118.83 (17)	H15A—C15—H15B	109.5
C10—C8—C7	123.55 (16)	C4—C15—H15C	109.5
O1—C9—C8	126.19 (18)	H15A—C15—H15C	109.5
O1—C9—H9A	116.9	H15B—C15—H15C	109.5
			
C9—O1—C1—C2	−178.25 (17)	C1—C6—C7—C8	−2.5 (3)
C9—O1—C1—C6	1.1 (3)	C5—C6—C7—C8	177.57 (16)
O1—C1—C2—C3	178.48 (17)	O2—C7—C8—C9	−177.91 (19)
C6—C1—C2—C3	−0.8 (3)	C6—C7—C8—C9	1.7 (2)
C1—C2—C3—C4	1.3 (3)	O2—C7—C8—C10	3.2 (3)
C2—C3—C4—C5	−1.4 (3)	C6—C7—C8—C10	−177.23 (16)
C2—C3—C4—C15	179.04 (19)	C1—O1—C9—C8	−2.1 (3)
C3—C4—C5—C6	0.9 (3)	C10—C8—C9—O1	179.58 (17)
C15—C4—C5—C6	−179.49 (19)	C7—C8—C9—O1	0.6 (3)
C2—C1—C6—C5	0.4 (3)	C9—C8—C10—C11	−179.17 (19)
O1—C1—C6—C5	−178.87 (16)	C7—C8—C10—C11	−0.2 (3)
C2—C1—C6—C7	−179.49 (17)	C8—C10—C11—C12	178.09 (17)
O1—C1—C6—C7	1.2 (3)	C13—O4—C12—O3	−1.0 (3)
C4—C5—C6—C1	−0.5 (3)	C13—O4—C12—C11	178.89 (16)
C4—C5—C6—C7	179.43 (17)	C10—C11—C12—O3	6.7 (3)
C1—C6—C7—O2	177.08 (18)	C10—C11—C12—O4	−173.18 (17)
C5—C6—C7—O2	−2.8 (3)	C12—O4—C13—C14	−172.99 (17)

### Hydrogen-bond geometry (Å, º)

**Table:**

*D*—H···*A*	*D*—H	H···*A*	*D*···*A*	*D*—H···*A*
C11—H11*A*···O2	0.93	2.28	2.908 (2)	124
C9—H9*A*···O3^i^	0.93	2.37	3.276 (3)	164

Symmetry code: (i) −*x*+1, −*y*+1, −*z*.
